# The perioperative outcomes of retroperitoneal lymph node dissection in Germany for patients with testicular cancer: Results from the GRAND study

**DOI:** 10.1002/ijc.35486

**Published:** 2025-06-04

**Authors:** Nikolaos Pyrgidis, Maria Apfelbeck, Marc Kidess, Philipp Weinhold, Julian Marcon, Gerald B. Schulz, Yannic Volz, Benedikt Ebner, Peter Maximilian Sparwasser, Igor Tsaur, Marcus Hentrich, Christian G. Stief, Michael Chaloupka

**Affiliations:** ^1^ Department of Urology University Hospital LMU Munich Munich Germany; ^2^ Department of Urology University Hospital Tübingen Tübingen Germany; ^3^ Department of Hematology and Oncology Red Cross Hospital LMU Munich Munich Germany

**Keywords:** mortality, perioperative outcomes, retroperitoneal lymph node dissection, robotic RPLND, testicular cancer

## Abstract

We aimed to assess the current trends and outcomes of retroperitoneal lymph node dissection (RPLND) in patients with testicular cancer in Germany, as well as to provide evidence on the role of the type of surgical approach, prior chemotherapy, and annual hospital caseload. We used the GeRmAn Nationwide inpatient Data, provided by the Research Data Center of the Federal Bureau of Statistics (2005–2022). We assessed trends and perioperative outcomes (mortality, intensive care unit [ICU] admission, transfusion, acute embolism, and length of hospital stay) based on the surgical approach (robotic, laparoscopic, and open), prior chemotherapy, and annual hospital caseload (with cut‐offs of three and 10 cases/year) with a multivariable regression analysis. Overall, 6673 patients underwent RPLND for testicular cancer. Of them, 5570 (83%) received open, 819 (12%) laparoscopic, and 284 (5%) robot‐assisted surgery. Patients had previously received chemotherapy in 1908 (29%) cases. Accordingly, 4431 (66%) patients underwent surgery in centers performing more than 3 cases/year, and 1325 (20%) in centers performing more than 10 cases/year. Over the past 18 years, the number of patients undergoing RPLND has decreased by half. In the multivariate regression analysis, a robotic and a laparoscopic approach was associated with lower odds of ICU admission, transfusion, and shorter hospital stay (*p* < .001) compared to an open approach. Patients undergoing surgery after prior chemotherapy presented similar perioperative outcomes compared to those who had not previously received chemotherapy. Similarly, patients undergoing surgery at high‐volume centers presented comparable perioperative outcomes to those in low‐volume centers based on the cut‐off of three and 10 cases/year. Still, our findings were mitigated by selection bias. Overall, the number of annual RPLND cases in Germany has decreased over time.

## What's new?

This nationwide analysis explores the recent trends and perioperative outcomes of retroperitoneal lymph node dissection for testicular cancer, stratified by surgical approach, prior chemotherapy, and hospital caseload. Since 2005, the number of patients undergoing retroperitoneal lymph node dissection for testicular cancer has decreased twofold in Germany. However, the use of robotic surgery has increased exponentially and is associated with shorter hospital stays. Patients undergoing surgery at low‐ and high‐volume centers, and those before and after chemotherapy, present comparable perioperative outcomes. The findings suggest that the indications of retroperitoneal lymph node dissection for testicular cancer have become more selective over time.

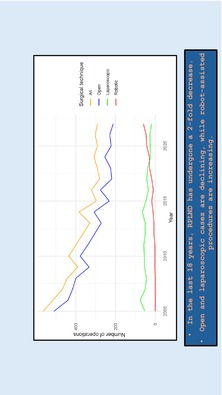

**Abbreviations**
CI: confidence interval
DRG: Diagnosis Related Groups
GRAND: GeRmAn Nationwide inpatient Data
ICU: intensive care unit
IQR: interquartile range
OR: odds ratio
RPLND: retroperitoneal lymph node dissection

## 1 INTRODUCTION

Malignant germ cell tumors of the testis are generally rare, representing 1% of adult neoplasms and 5% of urological tumors.^1^ Compared to other urological tumors, the peak incidence of testicular cancer is during the third and fourth decades of life.^2^ Moreover, most testicular malignant germ cell tumor cases are diagnosed at a localized stage and present an excellent long‐term prognosis.^3^ This means that avoiding the short‐ and long‐term complications of oncological therapy is of utmost importance.^4^

A plethora of treatment protocols have been suggested to reduce the complication rates of chemotherapy and radiotherapy used for testicular cancer.^5^ There is a recent shift toward primary retroperitoneal lymph node dissection (RPLND) in low‐volume metastatic disease over radiotherapy and chemotherapy.^6^ Indeed, chemotherapy and radiotherapy for metastatic testicular cancer have been associated with long‐term complications and secondary cancer.^7^ Thus, efforts are made in order to reduce the complication rates of RPLND in patients with metastatic germ cell tumors of the testis.^8^ In an attempt to optimize outcomes, a laparoscopic or robot‐assisted approach has been suggested.^9^ Similarly, accumulating evidence from other urological tumors suggests that patients might benefit from a centralized treatment strategy.^10^, ^11^ Nevertheless, studies comparing the perioperative outcomes of open versus laparoscopic versus robot‐assisted approaches are scarce. Similarly, studies aiming to define a clear cut‐off for sufficient expertise in terms of RPLND are lacking.

Within this scope, we aimed to assess the current trends and perioperative outcomes of RPLND in patients with testicular cancer, as well as to provide evidence on the role of the type of surgical approach, prior chemotherapy, and annual hospital caseload through the largest study in the field.

## 2 METHODS

### 2.1 Data source

For the present study, we utilized the GeRmAn Nationwide inpatient Data (GRAND) provided by the Federal Bureau of Statistics in Germany. The GRAND dataset encompasses comprehensive in‐hospital patient data from 2005 to 2022 within Germany, excluding military, psychiatric, and forensic cases. These data are maintained anonymized at the Research Data Center of the German Bureau of Statistics and were accessed for analysis under the agreement LMU—4710‐2022. GRAND includes information on comorbidities, surgical interventions, and perioperative outcomes. This information is coded according to the International Statistical Classification of Diseases and Related Health Problems, 10th revision, German modification (ICD‐10‐GM), and the German Procedure Classification (OPS). All data are limited to the inpatient period after the procedure, with no available information after patient discharge. Following the introduction of a diagnosis‐ and procedure‐related remuneration system in Germany (German Diagnosis Related Groups‐DRG) in 2004, all hospitals transmit these data to the Institute for the Hospital Remuneration System to receive their remuneration.

### 2.2 Selection criteria, coding, and annual hospital caseload threshold

We included all patients with testicular cancer (ICD‐10 code: C62) undergoing RPLND (OPS code: 5‐404.d or 5‐404.e). To obtain patient information on further comorbidities, surgical interventions, and perioperative outcomes, we used the available diagnostic and procedural codes (ICD‐10‐GM and OPS). A subgroup analysis was also performed based on the surgical approach (open [OPS code: 5‐404.d] vs. laparoscopic [OPS code: 5‐404.e] vs. robot‐assisted [OPS code: 5‐987]), as well as based on prior chemotherapy administration for testicular cancer (ICD‐10 code: Z08.2 or Z92.6).

The primary outcome of the present study was to assess the role of surgical approach (robot‐assisted vs. laparoscopic vs. open) in terms of perioperative outcomes (mortality, intensive care unit [ICU] admission, transfusion, acute embolism) and length of hospital stay. ICU admission was either elective after surgery or emergent due to perioperative complications. Secondary outcomes included the role of prior chemotherapy, as well as the surgical trends in RPLND for testicular cancer during the last 18 years in Germany. Subsequently, we evaluated the role of the annual hospital caseload on predicting perioperative outcomes. Based on previous studies on this topic, we defined a threshold of three and 10 RPLND per hospital per year.^12^ Hospitals were defined as high‐volume centers if they performed at least 10 RPLND for testicular cancer per year.^12^

### 2.3 Data synthesis and statistical analysis

Due to data anonymity, our research team was not permitted direct access to patient‐level data. Consequently, all statistical analyses were conducted by the Research Data Center of the Federal Bureau of Statistics using R scripts developed by our team (source: Research Data Center of the Federal Bureau of Statistics, DRG Statistics 2005–2022, own calculations). The resulting summary statistics were then provided to our team for further analysis. According to German legislation, approval from an ethics committee or patient informed consent was not required, given that we used administrative billing data.

All continuous variables were summarized as median with interquartile range (IQR) and all categorical variables as frequencies with proportions. The corresponding comparisons among robotic, laparoscopic, and open approaches were undertaken with the chi‐squared for categorical variables and the Kruskal–Wallis test for continuous variables. Accordingly, the comparisons between chemotherapy‐naïve and post‐chemotherapy patients, as well as between those operated in high‐volume centers versus low‐volume centers were undertaken with the chi‐squared for categorical variables and the Mann–Whitney test for continuous variables. We also performed a multivariable logistic and linear regression analysis to assess the perioperative outcomes (mortality, ICU admission, transfusion, acute embolism and length of hospital stay). All regression models were adjusted for age, obesity, hypertension, diabetes, history of chronic kidney disease, prior chemotherapy, surgical approach, and the year of operation. Odds ratios (ORs) with 95% confidence intervals (CIs) were calculated for all logistic models, with two‐sided *p*‐values <.05 considered statistically significant.

## 3 RESULTS

### 3.1 Baseline characteristics and trends

**FIGURE 1:**
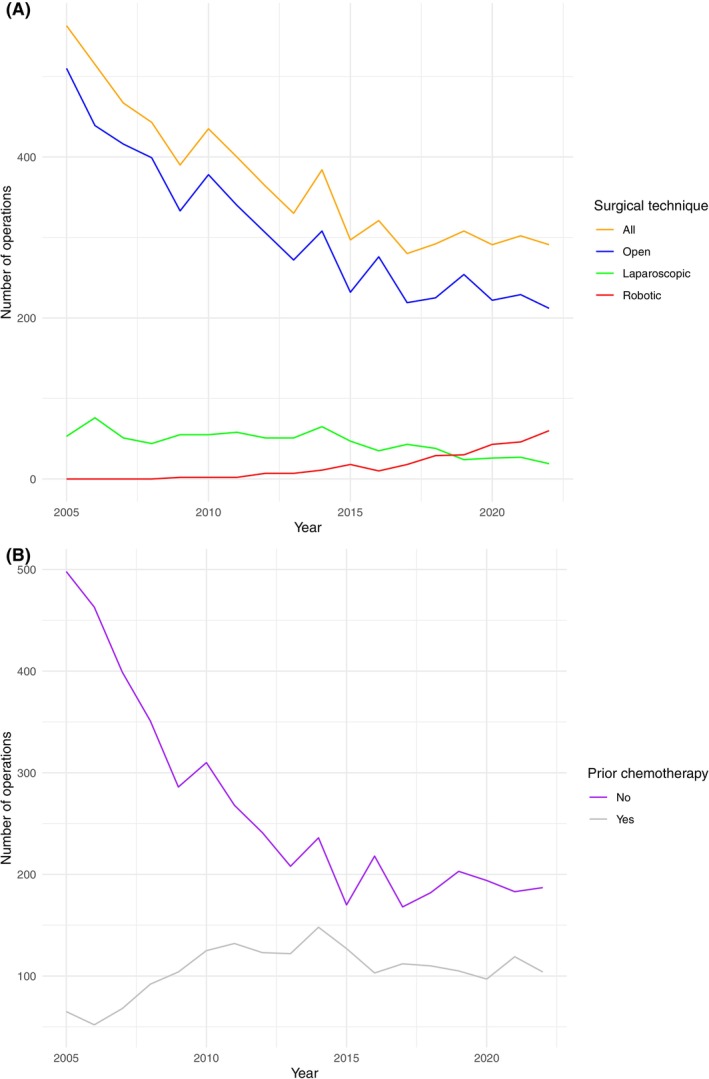
The annual trends for retroperitoneal lymph node dissection in patients with testicular cancer based on (A) surgical technique and (B) prior chemotherapy. [Color figure can be viewed at wileyonlinelibrary.com]

**FIGURE 2:**
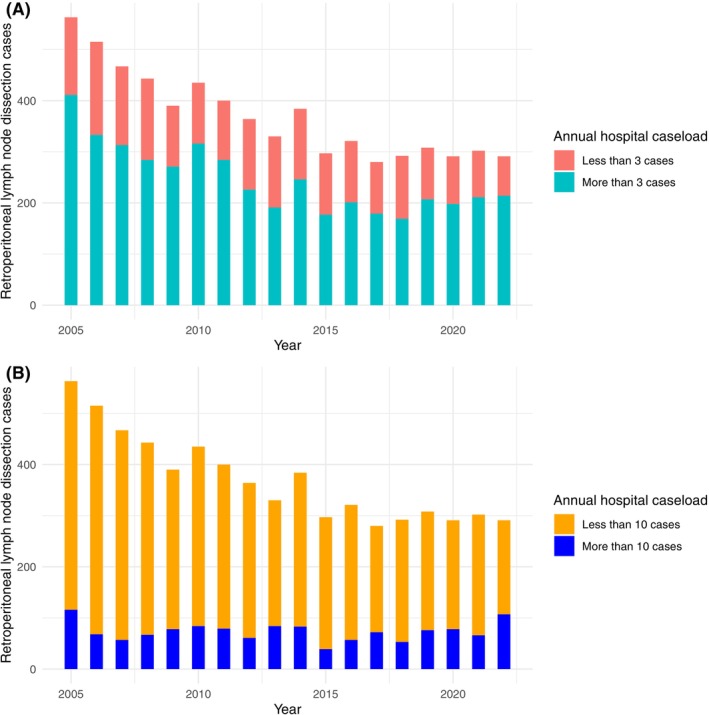
The annual trends for retroperitoneal lymph node dissection in patients with testicular cancer based on hospital caseload (A) less than three versus more than more annual cases and (B) less than 10 versus more than 10 annual cases. [Color figure can be viewed at wileyonlinelibrary.com]

A total of 6673 patients with a median age of 33 years (IQR: 26–42) underwent RPLND for testicular cancer from 2005 to 2022 in Germany. Of them, 5570 (83%) received open surgery, 819 (12%) laparoscopic surgery, and 284 (5%) robot‐assisted surgery (Table 1). Patients had previously received chemotherapy in 1908 (29%) cases. Accordingly, 4431 (66%) patients underwent surgery in centers performing more than 3 cases/year, and 1325 (20%) patients in centers performing more than 10 cases/year. In the last years, RPLND for testicular cancer has undergone an about two‐fold decrease from 563 cases in 2005 to 291 cases in 2022. Cases performed with an open or a laparoscopic approach are steadily decreasing, whereas those performed with a robot‐assisted approach are exponentially increasing. Furthermore, RPLND after prior chemotherapy has remained relatively stable, whereas cases undergoing RPLND without prior chemotherapy are continuously decreasing. The annual trends of RPLND for testicular cancer based on surgical technique and prior chemotherapy are presented in Figure 1, while the annual trends based on hospital caseload are illustrated in Figure 2.

**TABLE 1:** Baseline characteristics of the included patients undergoing retroperitoneal lymph node dissection for testicular cancer based on the surgical approach. Variables are presented as median with interquartile range or frequencies with proportions. The Kruskal–Wallis test was performed for comparisons between continuous variables and the chi‐squared test for categorical variables. The bold cells indicate statistically significant *p*‐values.

Characteristic	Overall, *n* = 6673	Open, *n* = 5570, 83%	Laparoscopic, *n* = 819, 12%	Robot‐assisted, *n* = 284, 5%	*p*‐Value
Age (years)	33 (26–42)	33 (26–42)	32.0 (26–41)	33 (27–42)	.48
Diabetes	135 (2%)	115 (2.1%)	15 (1.8%)	5 (1.8%)	.86
Chronic kidney disease	182 (2.7%)	170 (3.1%)	9 (1.1%)	3 (1.1%)	.**001**
Hypertension	604 (9.1%)	527 (9.5%)	55 (6.7%)	22 (7.7%)	.**028**
Obesity	352 (5.3%)	298 (5.4%)	41 (5.0%)	13 (4.6%)	.8
Year of surgery					
2005	563 (8.4%)	510 (9.2%)	53 (6.5%)	0 (0%)	
2006	515 (7.7%)	439 (7.9%)	76 (9.3%)	0 (0%)	
2007	467 (7.0%)	416 (7.5%)	51 (6.2%)	0 (0%)	
2008	443 (6.6%)	399 (7.2%)	44 (5.4%)	0 (0%)	
2009	390 (5.8%)	333 (6.0%)	—	Less than 3 cases	
2010	435 (6.5%)	378 (6.8%)	—	Less than 3 cases	
2011	400 (6.0%)	340 (6.1%)	—	Less than 3 cases	
2012	364 (5.5%)	306 (5.5%)	51 (6.2%)	7 (2.5%)	
2013	330 (4.9%)	272 (4.9%)	51 (6.2%)	7 (2.5%)	
2014	384 (5.8%)	308 (5.5%)	65 (7.9%)	11 (3.9%)	
2015	297 (4.5%)	232 (4.2%)	47 (5.7%)	18 (6.3%)	
2016	321 (4.8%)	276 (5.0%)	35 (4.3%)	10 (3.5%)	
2017	280 (4.2%)	219 (3.9%)	43 (5.3%)	18 (6.3%)	
2018	292 (4.4%)	225 (4.0%)	38 (4.6%)	29 (10%)	
2019	308 (4.6%)	254 (4.6%)	24 (2.9%)	30 (11%)	
2020	291 (4.4%)	222 (4.0%)	26 (3.2%)	43 (15%)	
2021	302 (4.5%)	229 (4.1%)	27 (3.3%)	46 (16%)	
2022	291 (4.4%)	212 (3.8%)	19 (2.3%)	60 (21%)	

### 3.2 Outcomes based on surgical technique

After adjusting for baseline parameters in the multivariate regression analysis (Table 2), a robotic approach, compared to an open approach, was associated with lower odds of ICU admission (8.1% vs. 25%; OR: 0.34, 95% CI: 0.21–0.52, *p* < .001), and transfusion (2.8% vs. 19%; OR: 0.14, 95% CI: 0.06–0.27, *p* < .001), as well as with shorter hospital stay (5 vs. 10 days; day difference: −3.6, 95% CI: −4.6 to −2.7, *p* < .001). Similarly, a laparoscopic approach, compared to an open approach, was also associated with lower odds of ICU admission (13% vs. 25%; OR: 0.45, 95% CI: 0.37–0.56, *p* < .001), and transfusion (3.2% vs. 19%; OR: 0.15, 95% CI: 0.1–0.21, *p* < .001), as well as with shorter hospital stay (6 vs. 10 days; day difference: −4, 95% CI: −4.5 to −3.4, *p* < .001). Overall, 30 (0.4%) deaths were observed. All patients that died during hospital stay had undergone open surgery. Accordingly, 70 (1%) patients had an acute embolism. Of them, 66 (1.2%) had undergone open surgery. Due to the low number of events (below three), further perioperative outcomes could not be assessed.

**TABLE 2:** Multivariable logistic and linear regression analysis for the effect of the surgical approach for retroperitoneal lymph node dissection on perioperative outcomes. The bold cells indicate statistically significant *p*‐values.

Outcome	Open		Laparoscopic		Robot‐assisted	
	Cases	Estimate (95% CI), *p*‐value	Cases	Estimate (95% CI), *p*‐value	Cases	Estimate (95% CI), *p*‐value
ICU admission	1388 (25%)	—	106 (13%)	OR: **0.45** (**0.37**, **0.56**), **<.001**	23 (8.1%)	OR: **0.34** (**0.21**, **0.52**), **<.001**
Transfusion	1047 (19%)	—	26 (3.2%)	OR: **0.15** (**0.10**, **0.21**), **<.001**	8 (2.8%)	OR: **0.14** (**0.06**, **0.27**), **<.001**
Length of hospital stay	10 (8–12)	—	6 (5–8)	Days: **−4** (**−4.5**, **−3.4**), **<.001**	5 (4–7)	Days: **−3.6** (**−4.6**, **−2.7**), **<.001**

Abbreviations: CI, confidence interval; ICU, intensive care unit; OR, odds ratio.

### 3.3 Outcomes based on prior chemotherapy administration

Patients undergoing RPLND for testicular cancer after prior chemotherapy, compared to those undergoing surgery without prior chemotherapy, presented similar perioperative outcomes in terms of mortality (*p* = .5), ICU admission (*p* = .6), acute embolism (*p* = .7) and length of hospital stay (*p* = .5). Only transfusion rates were higher in post‐chemotherapy versus chemotherapy‐naïve patients (20% vs. 15%; OR: 1.45, 95% CI: 1.25–1.68, *p* < .001). The multivariable analyses and the number of events are available in Table 3.

**TABLE 3:** Multivariable logistic and linear regression analysis for the effect of prior chemotherapy (no prior chemotherapy versus prior chemotherapy) and annual hospital caseload (less than 3 vs. more than 3 annual cases and less than 10 vs. more than 10 annual cases) for retroperitoneal lymph node dissection on perioperative outcomes. The bold cells indicate statistically significant *p*‐values.

Outcome	Prior chemotherapy		Centers with≥3 annual cases		Centers with≥10 annual cases	
	Cases	Estimate (95% CI), *p*‐value	Cases	Estimate (95% CI), *p*‐value	Cases	Estimate (95% CI), *p*‐value
Mortality	23 (0.5%) versus 7 (0.4%)	OR: 0.73 (0.28, 1.7), .5	14 (0.6%) versus 16 (0.4%)	OR: 0.6 (0.29, 1.28), .2	27 (0.5%) versus 3 (0.2%)	OR: 0.51 (0.12, 1.47), .3
ICU admission	1083 (23%) versus 434 (23%)	OR: 1 (0.91, 1.2), .6	475 (21%) versus 1042 (24%)	OR: **1.2** (**1.03**, **1.3**), **.02**	1259 (24%) versus 258 (19%)	OR: **0.81** (**0.69**, **0.94**), **.006**
Transfusion	700 (15%) versus 381 (20%)	OR: **1.45** (**1.25**, **1.68**), **<.001**	262 (12%) versus 819 (18%)	OR: **1.83** (**1.57**, **2.14**), **<.001**	813 (15%) versus 268 (20%)	OR: **1.46** (**1.25**, **1.72**), **<.001**
Acute embolism	49 (1.0%) versus 21 (1.1%)	OR: 0.91 (0.53, 1.52), .7	28 (1.2%) versus 42 (0.9%)	OR: 0.77 (0.48, 1.27), .3	50 (0.9%) versus 20 (1.5%)	OR: 1.54 (0.89, 2.58), .11
Length of hospital stay	9 (7–12) versus 9 (7–12)	Days: 0.13 (−0.28, 0.53), .5	9 (7–12) versus 9 (7–12)	Days: 0.08 (−0.3, 0.46), .7	9 (7–12) versus 8 (7–11)	Days: **−0.59** (**−1**, **−0.13**), **.01**

Abbreviations: CI, confidence interval; ICU, intensive care unit; OR, odds ratio.

### 3.4 Outcomes based on hospital caseload

Patients undergoing RPLND in centers performing more than three annual cases presented similar mortality (*p* = .2), acute embolism rates (*p* = .3) and length of hospital stay (*p* = .7) compared to those undergoing surgery in centers performing less than three annual cases. Interestingly, ICU admissions, either elective or emergent (24% vs. 21%; OR: 1.2, 95% CI: 1.03–1.3, *p* = .02) and transfusion rates (18% vs. 12%; OR: 1.83, 95% CI: 1.57–2.14, *p* < .001) were higher in patients undergoing surgery in centers performing more than three annual cases. Similarly, patients undergoing RPLND in centers performing more than 10 annual cases presented similar mortality (*p* = .3) and acute embolism rates (*p* = .1) compared to those undergoing surgery in centers performing less than 10 annual cases. Centers performing more than 10 annual cases were associated with lower ICU admissions (19% vs. 24%; OR: 0.81, 95% CI: 0.69–0.94, *p* = .006) and with shorter hospital stay (8 vs. 9 days; day difference: −0.59, 95% CI: −1 to −0.13, *p* = .01) but with higher transfusion rates (20% vs. 15%; OR: 1.46, 95% CI: 1.25–1.72, *p* < .001) compared to centers performing less than 10 annual cases (Table 3).

## 4 DISCUSSION

The findings of the present study indicate that the number of primary RPLNDs performed on chemotherapy‐naïve patients has been on a gradual decline over the past century, while the number of post‐chemotherapy RPLNDs has remained stable. The number of cases performed with an open or a laparoscopic approach undergoes a steady decrease. On the contrary, robotic surgery is exponentially increasing. Notably, the incidence of perioperative outcomes following RPLND in chemotherapy‐naïve patients and post‐chemotherapy patients was comparable. Nevertheless, the number of chemotherapy‐naïve patients undergoing surgery is continuously decreasing.

Although RPLND represents a fundamental surgical intervention in the treatment of advanced testicular cancer, the range of its indications has narrowed over time. As an adjuvant treatment of non‐seminomatous clinical stage 1 testicular cancer, RPLND has been replaced by risk‐adapted strategies with either surveillance or platinum‐based chemotherapy due to a lower risk of relapse and lower reproducibility of RPLND compared to chemotherapy on a large scale.^13^ Only for high‐risk patients with clinical Stage 1 non‐seminomatous testicular cancer and a somatic malignant component who are unfit or unwilling to undergo adjuvant chemotherapy, RPLND may be offered in centers of expertise.^14^ For patients with clinical Stage 2 seminomatous testicular cancer, several therapeutic options are available: chemotherapy, radiotherapy, and primary RPLND. Multicenter trials show promising results with the combination of radiotherapy and chemotherapy.^4^ The role of primary RPLND in this setting is controversial. Meanwhile, studies on RPLND in clinical Stage 2 testicular cancer report a large variety in surgical technique/template, patient selection, adjuvant chemotherapy, and length of follow‐up, which collectively render direct comparisons challenging. The PRIMETEST trial did not meet its primary endpoint of progression‐free survival of <30% at 36 months.^15^ The retrospective evaluation of 45 patients by colleagues at the MSKCC by Matulewicz et al. showed an overall low complication rate of primary RPLND. The postoperative observation strategy in 29 patients resulted in a 2‐year recurrence‐free survival of 81%, sparing the majority of patients from chemotherapy or radiotherapy with possible long‐term consequences.^16^ The decreasing use of primary RPLND is also evident in our analysis, with the number of RPLND in chemotherapy‐naïve patients decreasing by more than 100% in the last decade. However, our analysis does not provide detailed information on the seminomatous or non‐seminomatous origin of the analyzed testicular cancer patients.

RPLND is a demanding procedure with high complication rates.^17^ Available evidence suggests that RPLND in patients who have previously undergone chemotherapy is more challenging.^18^ This is predominantly attributed to the desmoplastic reaction of residual tumors on adjacent structures and the impaired pulmonary, renal, and hematologic reserves of these patients.^18^ However, it seems that perioperative outcomes do not differ between chemotherapy‐naïve and post‐chemotherapy patients.^19^ These findings are in line with our results. In particular, acute pulmonary embolism, ICU admission, and mortality did not differ between the two groups. However, the transfusion rates were significantly higher in post‐chemotherapy patients. This might be attributed to the compromised hematologic reserves even in younger patients with a history of chemotherapy.^20^

Current guideline recommendations suggest that RPLND should be performed at high‐volume centers as it may be associated with improved short‐ and long‐term outcomes.^21^ Nevertheless, there is no evidence‐based threshold to distinguish high‐ from low‐volume centers. A minimum of three RPLND per year per surgeon is required for certification as a Center for Testicular Cancer by the German Cancer Society.^22^ Still, when applying this threshold, our analysis showed that mortality, pulmonary embolism, and length of hospital stay were similar between hospitals with a caseload of more and less than three cases per year. Interestingly, elective or emergent ICU admissions and transfusion rates were even higher in centers performing more than three cases annually. When applying a threshold of 10 RPLND per year as suggested by other studies,^12^ we observed higher transfusion rates but lower ICU admissions in hospitals with a caseload of more than 10 cases per year compared to hospitals with less than 10 cases per year. This inconsistency is influenced by the selective and individualized indication of this demanding procedure and illustrates that the sheer hospital caseload is an insufficient indicator of better perioperative outcomes of RPLND.

Therefore, it seems that the surgical approach may play a key role in reducing the perioperative complication rates.^23^ Our findings may indicate better perioperative outcomes after a minimally invasive approach compared to open surgery. In particular, a robotic approach was associated with lower odds of ICU admission, transfusion, and shorter hospital stay compared to open surgery. Due to the low number of events after a minimally invasive approach, further perioperative outcomes could not be evaluated. Still, it should be highlighted that our study is solely based on epidemiological data and was not designed to explore definitive causal relationships for the different surgical approaches. RPLND is a highly demanding surgical procedure whose execution depends on factors such as tumor stage, localization, and the experience of the treating clinic. Since these variables are not documented in our epidemiological data, it is not possible to establish a causal relationship to determine which surgical method is the safest. In a retrospective study by Lloyd et al. analyzing 28 post‐chemotherapy patients treated by robot‐assisted RPLND versus 72 patients treated by open RPLND, the robot‐assisted approach demonstrated lower perioperative blood loss and shorter length of hospital stay.^24^ Similarly, comparing robot‐assisted RPLND versus laparoscopic and open RPLND in chemotherapy‐naïve patients, Lin et al. also showed lower blood loss and shorter length of hospital stay in patients undergoing robot‐assisted RPLND.^25^ Still, although the robot‐assisted RPLND was associated with promising results, higher rates of postoperative lymphoceles and tumor recurrence have been reported.^26^

Even though we report, to the best of our knowledge, the first study focusing on the trends and perioperative outcomes of RPLND for testicular cancer based on the surgical approach, prior chemotherapy, and annual hospital caseload, our findings are not devoid of some limitations that need to be underlined. Firstly, our analyses derive from administrative billing data and are, therefore, prone to coding errors and misclassifications. Even though these data are regularly assessed by independent medical task forces, important information is still missing. In particular, the type of histology (seminoma vs. non‐seminomatous germ cell tumor), the patient's laboratory findings, the type and duration of prior chemotherapy, the operative time, and the oncological status (TNM classification, extent and location of lymph node infiltration, and surgical margins) are not available in the GRAND study. Based on the previous notion, we fully acknowledge that patient selection plays a crucial role in determining the surgical approach, with minimally invasive techniques often being reserved for patients with lower disease burden, whereas open surgery remains the standard in more advanced or complex cases. Therefore, our findings predominantly describe associations and are hypothesis‐generating. Of note, data on perioperative outcomes after hospital discharge, readmission, and reoperation rates, as well as long‐term follow‐up data, are not collected in the GRAND study.

There is a plethora of important factors that we could not measure or include in our regression modeling that may have affected the relationship between surgical approach and perioperative outcomes, including the complexity of the procedure, the experience of the surgeon, the fitness of the patient, his body mass index, and socioeconomic status. It should also be noted that the outcomes related to ICU admission may not reflect the real outcomes, since it may be standard for all patients in some hospitals who undergo open procedures to automatically go to the ICU following surgery, so that they can be carefully monitored. Furthermore, our findings focus on the annual hospital caseload and not on the surgeon caseload. Importantly, the Federal Bureau of Statistics excluded all outcomes with fewer than three cases to ensure anonymity. Therefore, further analyses could not be performed. It should be highlighted that our findings may present limited generalizability, given that they derive solely from Germany. Similarly, patients selected for a robotic approach or patients operated on in high‐volume centers often differ from those selected for an open approach or from those operated on in low‐volume centers in terms of comorbidities and surgical indications. Therefore, the better perioperative outcomes observed after robot‐assisted surgery and the inconsistency of the perioperative outcomes in low‐ versus high‐volume centers may be the result of selection bias.

## 5 CONCLUSION

The present nationwide inpatient data suggest that the indications of RPLND for testicular cancer have become more selective over time. In recent years, there has been a noticeable decline in the number of chemotherapy‐naïve patients undergoing surgery, suggesting a preference for chemotherapy or radiotherapy before considering RPLND. Notably, our analysis demonstrates that prior chemotherapy does not affect perioperative outcomes after RPLND. Furthermore, higher annual hospital caseload was not associated with improved outcomes. Still, the present study is limited by its retrospective, epidemiological design, which is inherently susceptible to selection bias.

## AUTHOR CONTRIBUTIONS

**Nikolaos Pyrgidis:** Writing – original draft; formal analysis. **Maria Apfelbeck:** Conceptualization; methodology. **Marc Kidess:** Software; validation. **Philipp Weinhold:** Writing – review and editing; visualization. **Julian Marcon:** Writing – review and editing; project administration. **Gerald B. Schulz:** Supervision; resources; investigation. **Yannic Volz:** Methodology; writing – review and editing. **Benedikt Ebner:** Methodology; writing – review and editing. **Peter Maximilian Sparwasser:** Validation; writing – review and editing. **Igor Tsaur:** Validation; writing – review and editing. **Marcus Hentrich:** Writing – review and editing; methodology. **Christian G. Stief:** Investigation; funding acquisition; writing – review and editing. **Michael Chaloupka:** Conceptualization; writing – original draft; project administration; resources.

## CONFLICT OF INTEREST STATEMENT

The authors declare no conflicts of interest.

## ETHICS STATEMENT

Written informed consent from the participants, as well as ethical approval, was not required for the present study following the national legislation and institutional requirements. All data used in this work are stored anonymized at the German Federal Statistical Office.

## Data Availability

Data from the GeRmAn Nationwide inpatient Data (GRAND) provided by the Federal Bureau of Statistics, Germany was used for this study. Further (deidentified) information is available from the corresponding author upon reasonable request.
